# Evaluation of a Lung Ultrasound Score in Hospitalized Adult Patients with COVID-19 in Barcelona, Spain

**DOI:** 10.3390/jcm13113282

**Published:** 2024-06-02

**Authors:** Maria S. Lightowler, Julia Verena Sander, Gonzalo García de Casasola Sánchez, Maria Mateos González, Robert Güerri-Fernández, Maria Dolores Lorenzo Navarro, Fabienne Nackers, Erin Stratta, Candelaria Lanusse, Helena Huerga

**Affiliations:** 1 Epicentre, 75019 Paris, France; 2 Médecins Sans Frontières, 08005 Barcelona, Spain; 3 Hospital Infanta Cristina, 28981 Madrid, Spain; 4 Hospital del Mar, 08003 Barcelona, Spain; 5 Médecins Sans Frontières, New York, NY 10006, USA

**Keywords:** COVID-19, SARS-CoV-2, SpO_2_/FiO_2_ ratio, clinical outcome, lung ultrasound, scoring system

## Abstract

**Background/Objectives**: During the COVID-19 pandemic and the burden on hospital resources, the rapid categorization of high-risk COVID-19 patients became essential, and lung ultrasound (LUS) emerged as an alternative to chest computed tomography, offering speed, non-ionizing, repeatable, and bedside assessments. Various LUS score systems have been used, yet there is no consensus on an optimal severity cut-off. We assessed the performance of a 12-zone LUS score to identify adult COVID-19 patients with severe lung involvement using oxygen saturation (SpO_2_)/fractional inspired oxygen (FiO_2_) ratio as a reference standard to define the best cut-off for predicting adverse outcomes. **Methods**: We conducted a single-centre prospective study (August 2020–April 2021) at Hospital del Mar, Barcelona, Spain. Upon admission to the general ward or intensive care unit (ICU), clinicians performed LUS in adult patients with confirmed COVID-19 pneumonia. Severe lung involvement was defined as a SpO_2_/FiO_2_ ratio <315. The LUS score ranged from 0 to 36 based on the aeration patterns. **Results**: 248 patients were included. The admission LUS score showed moderate performance in identifying a SpO_2_/FiO_2_ ratio <315 (area under the ROC curve: 0.71; 95%CI 0.64–0.77). After adjustment for COVID-19 risk factors, an admission LUS score ≥17 was associated with an increased risk of in-hospital death (OR 5.31; 95%CI: 1.38–20.4), ICU admission (OR 3.50; 95%CI: 1.37–8.94) and need for IMV (OR 3.31; 95%CI: 1.19–9.13). **Conclusions**: Although the admission LUS score had limited performance in identifying severe lung involvement, a cut-off ≥17 score was associated with an increased risk of adverse outcomes. and could play a role in the rapid categorization of COVID-19 pneumonia patients, anticipating the need for advanced care.

## 1. Introduction

Coronavirus disease 2019 (COVID-19) pneumonia, caused by severe acute respiratory syndrome coronavirus 2 (SARS-CoV-2), presents a broad spectrum of clinical severity [1]. Approximately 14% of individuals infected with SARS-CoV-2 experienced severe respiratory illness, necessitating hospitalization and oxygen therapy, and 5% were in critical condition [2]. Given the burden on hospital resources during the pandemic, and the diverse progression of patients, from those needing minimal care to others progressing to respiratory failure and intensive care unit (ICU) admission [3,4], it was essential to rapidly identify and categorize the patients with COVID-19 at high risk of severe disease.

Chest computed tomography (CT) constitutes a reference imaging technique for assessing the severity of lung involvement in COVID-19 patients [5]. However, it has limitations: CT scans may not be universally accessible, it poses risks during intrahospital transport [6], it can overwhelm resources, and it can increase the risk of infection transmission [7]. During the pandemic, lung ultrasound (LUS) was extensively used as an alternative technique [8]. It is a faster and non-ionizing method that allows for real-time and repeated assessments at the bedside. Furthermore, LUS appears to be a more viable option when a health system is under pressure from a pandemic and a more affordable option in settings with limited resources, such as low- and middle-income countries (LMICs).

Several LUS scoring systems measuring aeration patterns have been suggested, to guide clinicians in patient care decision-making. Originally used in patients with acute respiratory distress syndrome (ARDS), a 12-zone LUS score has demonstrated a correlation with disease severity and mortality prediction [9,10,11]. In 2020, this LUS score was applied to COVID-19 patients [12,13]. Various LUS zone protocols have been proposed, including 8-zones [14], 10-zones [15], 12-zones [13], and 14-zones [12]. The 12-zone protocol had been previously validated in various pathologies [16], appeared feasible for clinicians with limited experience in performing ultrasound [17], and, hence, appeared as a suitable option for COVID-19 patients. Subsequent studies have indicated that the 12-zone protocol strikes a good balance between diagnostic accuracy and acquisition time [16,18].

Some studies have shown that LUS score has a significant correlation with oxygen saturation (SpO_2_) and the fraction of inspired oxygen (FiO_2_) ratio, as well as the use of mechanical ventilation in patients with COVID-19 pneumonia [19,20]. Various published studies have looked at specific LUS cut-offs for mortality [21,22,23,24,25] and adverse outcomes [26,27,28]. However, a definitive consensus on the optimal cut-offs has not yet been reached [29].

In order to guide the clinical management of adult COVID-19 patients, we aimed to identify the best cut-offs for predicting hospital adverse outcomes. Initially, we assessed the 12-zone LUS score in determining severe lung involvement, with SpO_2_/FiO_2_ ratio serving as the reference standard. Subsequently, we aimed to define the best LUS score cut-off for predicting in-hospital mortality, the need for invasive mechanical ventilation (IMV), and admission to the ICU.

## 2. Materials and Methods

### 2.1. Study Design and Setting

We conducted a single-center prospective observational diagnostic study at the Hospital del Mar (HDM), in Barcelona, Spain. The HDM, with 400 beds and 41 beds for critical care, serves a very diverse and multicultural population. The HDM has been involved in the care and management of COVID-19 patients since the beginning of the epidemic in Catalunya.

### 2.2. Study Population and Recruitment

Recruitment and admission took place from 17 August 2020 to 29 April 2021, in the emergency room (ER), the medical ward, the intermediate care ward, and the ICU. The latest discharge from the hospital for the included patients occurred on 30 July 2021. The number of daily inclusions was adjusted to the workload of the study clinicians by selecting a daily sample of eligible patients using a systematic random procedure.

All admitted patients aged ≥18 years with respiratory symptoms on admission and a laboratory confirmed COVID-19 diagnosis were eligible for the study. The inclusion criteria were as follows: one, if they had at least one positive result by real-time reverse transcription polymerase chain reaction (RT-PCR) assay for SARS-CoV-2 on a nasopharyngeal swab sample; and two, if they had pneumonia, suspected by the presence of fever and at least one sign/symptom of lower respiratory tract infection such as cough, shortness of breath or chest pain, or confirmed by an infiltrate on the chest X-ray with no alternative diagnosis. The following were the exclusion criteria: Patients requiring home oxygen therapy due to severe chronic obstructive pulmonary disease (including emphysema, chronic bronchitis, and chronic obstructive asthma) or another severe pulmonary pathology like interstitial lung disease, patients with extensive pulmonary malignant disease and who are under palliative care, or patients not able to tolerate LUS examination. Patients were followed up until the end of hospitalization.

### 2.3. Study Procedures, LUS Examination and Scoring

Upon admission, eligible patients who provided informed consent were included in the study and received a LUS examination. All LUS scans and scoring were performed by clinicians or intensivists in charge of the patients. Eight clinicians did not have previous experience in ultrasound and received a ten-hour training (two hours of theoretical training, and eight hours of hands-on training) and five supervised scans. Two clinicians had experience in performing LUS with an Extended Focused Assessment with Sonography in Trauma certification (EFAST) and received the same training. One intensivist had extensive experience in performing LUS scans.

Trained clinicians were instructed on a protocol to perform a structured and systematic evaluation of the lungs and to assess their level of aeration. Bedside LUS was performed using a Philips Lumify portable tablet-based ultrasound device (Philips medical), with either a curvilinear or linear probe, with a frequency range of 2 to 5 MHz and 4 to 12 MHz, respectively. The depth was set to 8 cm on the linear probe and 12 cm on the curved probe, d. A specific standardized procedure outlined the selection and utilization of probes, with a preference for a linear transducer. To ensure thorough image evaluation, it was necessary for the distance between the skin and pleura to be visible a minimum of 3 times, thereby allowing for a minimum of 2 A-lines to be assessed. Considering the linear probe’s maximum depth capability of 7 cm, patients with a chest wall measuring less than 2.3 cm were assessed using this specific probe. Additionally, novice ultrasound users received supervision during their initial scans to guarantee accurate probe selection and adherence to the protocol. LUS scans were performed within the first 24–48 h after admission, and then up to 4 scans were performed every 48–72 h. Scans of 12 zones of the thorax were performed: the upper and lower parts of the anterior, lateral, and posterior regions of the left and right chest. Scans were performed along anatomical lines: mid-clavicular, mid-axillary, mid scapular on right and left side. For each line a clip of ten seconds was obtained, and each zone was assigned a score according to its aeration pattern (4 patterns, from 0 to 3 points: A-lines = 0 points, more than 3 B-lines = 1 point, confluent B-lines = 2 points, and consolidation = 3 points). The total LUS score was estimated by adding the scores of all the 12 zones (range of total score: 0 to 36) [13]. A score of 0 represents a normal lung and a score of 36 demonstrated the worst grade of severity. The clinician recorded the score of each zone and final score in a case report form. Stored images were saved and digitally forwarded for remote expert review.

The digital LUS images/videos were reviewed by two off-site LUS experts blinded to the clinical status and treatment of the patient and to the first scan score. The experts were two clinicians who used POCUS in their daily practice for several years. LUS scores obtained from experts were also recorded and used for the main analysis of the study.

### 2.4. Demographics and Clinical Information

Demographics and clinical information were systematically collected from the medical records including age, gender, comorbidities, symptoms, body temperature, respiratory rate, blood pressure, COVID-19 test result, routine laboratory findings, radiological results (X-ray and/or CT), date of ICU transfer if needed, type of required oxygen therapy, final clinical outcome, and date of death or discharge. Oxygen saturation (SpO_2_) and fraction of inspired oxygen (FiO_2_) were measured and noted at the time of LUS or retrieved from the medical record collected closest to the LUS time. The severity of lung involvement was categorized as per the level of hypoxemia. Peripheral oxygen saturation was measured by a pulse oximeter. A conversion table for the estimation of FiO_2_ provided by oxygen delivery devices was used. We used SpO_2_/FiO_2_ ratios of 235 and 315, which have been reported to correlate with PO_2_/FiO_2_ ratios of 200 and 300, respectively, in diagnosing and following lung injury and ARDS [30]. For the purpose of the analysis, we defined a SpO_2_/FiO_2_ ratio less than 315 as severe and less than 235 as very severe [31]. A basic cardiac point-of-care ultrasound (POCUS) examination was conducted upon admission using a cardiac probe and preset. A comprehensive echocardiogram was performed if a cardiac pathology was suspected.

### 2.5. Data Analysis and Sample Size

Continuous variables were presented as medians and interquartile ranges [IQR] and categorical variables as frequencies (percentages).

A receiver operating characteristic (ROC) curve was plotted to assess the performance of the expert LUS score at admission to identify patients with a SpO_2_/FiO_2_ ratio below 315. The area under the curve (AUC) was computed with 95% confidence intervals (95% CI). In addition, sensitivity, specificity, and positive (PPV) and negative predictive values (NPV) were calculated for several expert LUS score cut-offs, using SpO_2_/FiO_2_ as the reference standard, to assess the performance of each cut-off and identify patients with severe lung involvement.

Scatter plots, fitted with a locally weighted scatterplot smoothing (LOWESS) procedure, allowed for the visualising of correlations between adverse outcomes (in-hospital death, ICU admission, need for IMV, need for non-invasive mechanical ventilation [NIMV]) and the expert LUS score at admission or the difference between the expert scores of the first and the second LUS examinations. We selected several cut-off values after evaluating the LUS score performance, using SpO_2_/FiO_2_ as the reference standard and visualising correlations with LOWESS. We used univariate models to assess the association between the selected LUS score cut-offs and adverse hospital outcomes, evaluating each of them, and a combination of them (in-hospital death or ICU admission or need for IMV). Patients already in the ICU or receiving IMV or NIMV at the time of the LUS examination were excluded from the analysis. We used a multivariate logistic regression model, with the optimal cut-off point adjusted for a priori identified risk factors: age, gender (male), and the presence of hypertension, diabetes, and smoking status [32]. We also estimated the AUC, sensitivity, specificity, and PPV and NPV values (with 95% CI) of a LUS score ≥ 17 to identify any of the adverse outcomes.

Additionally, we performed a Bland–Altman analysis between a normalized inverted LUS score and normalized SpO_2_/FiO_2_ in the presence of any of the adverse outcomes (in-hospital death or ICU admission or need for IMV).

Inter-observer variability between the clinician and expert LUS scores was examined by Bland–Altman analysis.

All *p*-values are two-sided, with values below 0.05 considered significant. Data were entered into a REDCAP (Research electronic data capture) database and analyzed using Stata version 16.1 (College Station, TX, USA). Missing data were checked for each variable, and individuals with missing values were labelled as ‘‘unknown” and excluded from the related analyses.

A sample size of 252 patients was calculated using the following hypothesis: a LUS score cut-off expected to detect severity using SpO_2_/FiO_2_ levels as reference, with 80% sensitivity and 75% specificity, an alpha error of 0.05, a precision of +−8%, an expected prevalence of 50% of severe cases among COVID-19 admitted patients, and a 10% increase, allowed us to account for possible losses during follow-up or refusals.

### 2.6. Ethics Considerations

The study protocol was approved by the Hospital del Mar ethics committee (approval number 2020/9403/I) and by the MSF Ethics Review Board (approval number 2045). Patient informed consent was obtained before enrolment in the study. To limit the risk of coronavirus SARS-CoV-2 transmission from manipulating papers and pens, participants provided verbal informed consent.

## 3. Results

**Figure 1:**
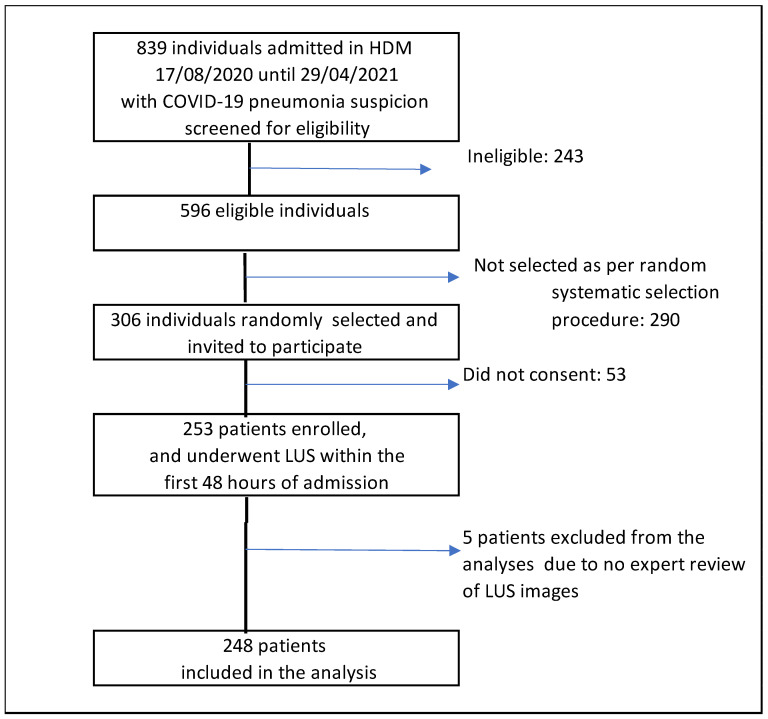
Study flow chart.

Out of the 839 patients admitted and screened for eligibility, 253 were enrolled and 248 were included in the analysis (Figure 1). All participants had a SARS-CoV-2 infection confirmed by real-time RT-PCR. The median age was 60 years (IQR: 51–73) and 151 (61.6%) were men. Most participants (247; 99.6%) underwent a chest X-ray and 60 (24.2%) a chest CT scan. Demographic characteristics, comorbidities, laboratory results, radiological findings, and outcomes are detailed in Table 1. Thirteen patients (5.2%) died after a median hospitalization duration of 23 days (IQR: 6–91). The remaining patients were discharged with a median hospitalization duration of 8.5 days (IQR: 6–15). Thirty-six patients (14.5%) required admission to the ICU, with ten admitted directly upon arrival, and 23 patients (9.3%) needed IMV, two of whom were already intubated at admission.

Trained clinicians and expert reviewers scored a total of 657 LUS examinations (1 to 5 LUS per patient), including 248 LUS examinations performed upon admission and 212 LUS examinations during hospitalization. The curvilinear probe was predominantly used, as the linear probe did not provide sufficient depth for accurate LUS scores on most patients. The median score was 11 (IQR: 7–15) at the initial LUS examination and 10 (IQR: 7–14) at the second LUS examination (Table 2).

Upon admission, 96 patients (38.7%) had a SpO_2_/FiO_2_ ratio <315, categorized as severe. The initial cardiac POCUS examination revealed a cardiac pathology in 21 patients (8.4%).

### 3.1. Performance of the LUS Score to Identify Patients with Severe Lung Involvement

**Figure 2:**
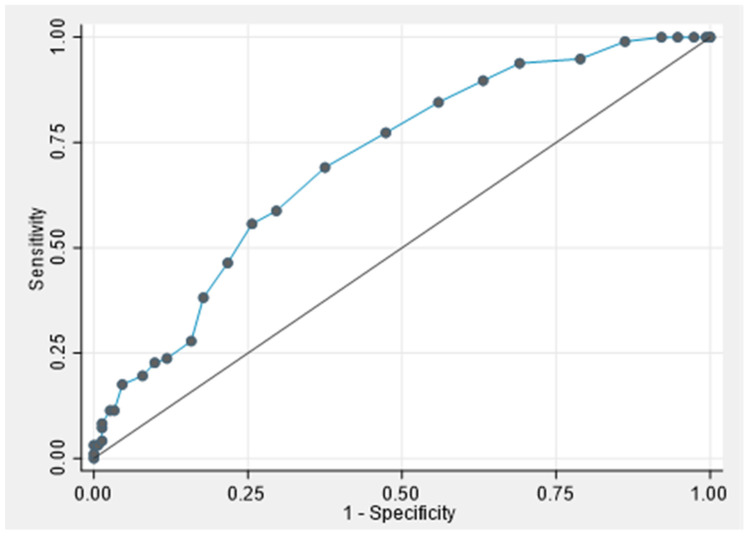
Receiver operating characteristic (ROC) curve assessing the performance of the expert lung ultrasound score at admission to identify a SpO_2_/FiO_2_ ratio <315 (*n* = 248), LUS score (dotted line, AUC: 0.71; 95% CI: 0.64–0.77).

The ROC curve analysis showed moderate performance of the expert LUS score upon admission in assessing the severity of the lung involvement (defined as SpO_2_/FiO_2_ ratio <315, *n* = 248), with an AUC of 0.71 (95% CI: 0.64–0.77) (Figure 2).

The sensitivity and specificity of various LUS score cut-offs to differentiate patients with a SpO_2_/FiO_2_ ratio <315 from other patients are presented in the Supplementary Table S1. A LUS score ≥7 at admission showed a sensitivity of 94% (95% CI: 91–97%) with a specificity of 30% (95% CI: 25–37%), a PPV of 46% (95% CI: 40–53%) and a NPV of 89% (95% CI: 85–93%). A LUS score ≥17 at admission (*n* = 41; 16.5%) showed a specificity of 88% (95% CI: 84–92%), with a sensitivity of 24% (95% CI: 18–29%), a PPV of 56% (95% CI: 50–62%) and an NPV of 64% (95% CI: 58–70). In total, 52 (20.9%) patients had a LUS score <7 and 41 (16.5%) a LUS score ≥17.

The Bland–Altman analysis results, between normalized inverted LUS score and normalized SpO_2_/FiO_2_ in the presence of any of the adverse outcomes (in-hospital death or ICU admission or need for IMV), are presented in Supplementary Figure S4.

### 3.2. Association between LUS Score and Adverse Hospital Outcomes

Univariate logistic regression models showed an association between several high LUS score cut-offs, including ≥17 (Table 3) and hospital adverse outcomes (increased risk of in-hospital death, ICU admission, need for IMV, need for NIVM, and combined adverse outcomes) (Supplementary Table S2). We selected the cut-off ≥17 based on the good specificity previously described, and because it was significantly associated with all the adverse outcomes in the univariate analysis. We presented the results of AUC, sensitivity, specificity, PPV and NPV in the Supplementary Table S3.

After adjustment, using the known COVID-19 risk factors for severity (age, gender, hypertension, diabetes, and smoking), an admission LUS score ≥17 was associated with an increased risk of in-hospital death (OR 5.31; 95% CI: 1.38–20.4; *p* = 0.015), ICU admission (OR 3.50; 95% CI: 1.37–8.94. *p* = 0.009), the need for IMV (OR 3.31; 95% CI: 1.19–9.13; *p* = 0.020) and the need for NIMV (OR 3.77; 95% CI: 1.69–8.40; *p* = 0.001) (Table 3).

LOWESS scatter plots examining the correlation between adverse hospital outcomes (and either the SpO_2_/FiO_2_ ratio, the expert LUS score upon admission, or the difference between the scores of the first and second LUS examinations) are shown in the Supplementary Figures. As the SpO_2_/FiO_2_ ratio decreased from 315, the curves suggested an increased risk of ICU admission, the need for IMV, the combined adverse outcomes (Supplementary Figure S1) and the need for NIMV (Supplementary Figure S2), but not of in-hospital death. When the LUS scores at admission ranged from 8 to 24, the curves showed an increased risk of the same adverse outcomes as the LUS scores at admission increased. A trend towards an increased risk of in-hospital death was observed when admission LUS scores exceeded 16. The difference between the first and second LUS scores showed no association with adverse hospital outcomes (Supplementary Figure S3).

### 3.3. LUS Score Inter-Observer Agreement

**Figure 3:**
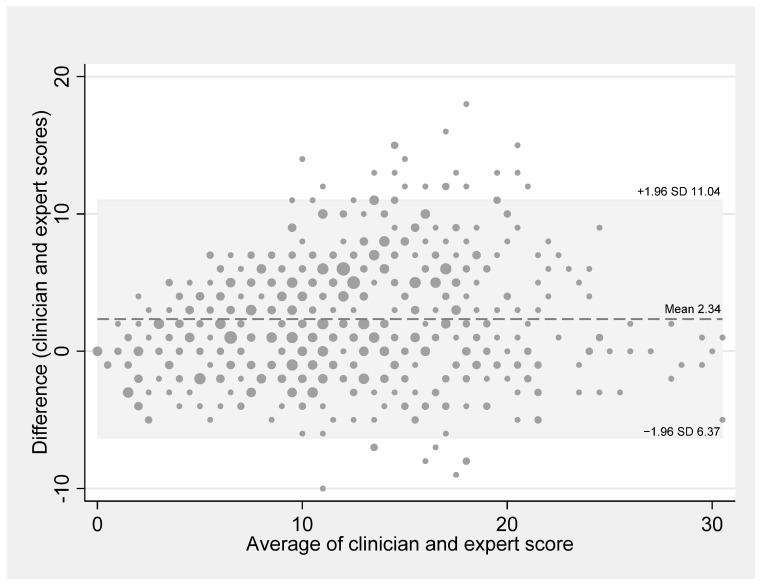
Bland–Altman plot comparing the lung ultrasound scores obtained by trained clinicians and by the off-site expert reviewers. The line indicates the mean difference (score from clinician—score from expert) and the grey area covers the 95% limits of agreement interval (mean difference ± 1.96 times standard deviation).

The Bland–Altman analysis (*n* = 643) showed a mean LUS score difference (trained clinicians—experts) of +2.34 (95% agreement interval: −6.4 to 11.0), with 27 (4.2%) data points falling outside the limits of agreement. The difference between the trained clinician and expert findings tended to be larger between LUS scores of 10 and 21 (Figure 3). Experts described B-lines as confluent less often, and a lower size of consolidations between the scores of 10 and 20 could be seen. However, when the scores tended to be low (<10) or high (>20), they tend to agree.

## 4. Discussion

We assessed the performance of a 12-zone LUS score measured upon hospital admission to identify severe lung involvement in COVID-19 patients with pneumonia. In our study, we found moderate performance of the LUS scoring system to identify patients with severe lung involvement, and no single LUS cut-off value offered a good trade-off between sensitivity and specificity to categorize patients according to their severity. However, a high LUS score at admission was associated with an increased risk of adverse outcomes during hospitalization.

Previous studies in various settings have reported that LUS scores inversely correlate with either the SpO_2_/FiO_2_ or PaO_2_/FiO_2_ ratio as a measure of respiratory failure or desaturation in COVID-19 patients [19,20,29,33]. In our study, a LUS score cut-off value ≥7 upon admission identified severe lung involvement with good sensitivity and NPV, but low specificity. This value could be useful for ruling out severe lung involvement in COVID-19 patients with a LUS score <7 upon admission, who constituted approximately a quarter of all patients. A LUS score cut-off value ≥17 upon admission had higher specificity but poor sensitivity, and allowed for the identifying of severe lung involvement in approximately a sixth of all patients. These findings may enable clinicians to categorize patients upon admission according to severity levels. Patients with a LUS score <7 could be classified as having mild severity, requiring standard care. Conversely, those with a LUS score ≥17 may benefit from more intensive monitoring or earlier oxygen therapy intervention. These results align with those observed in another hospital center in Spain [18].

Several studies have found a significant association between the LUS score and ICU admission, the need for mechanical ventilation, and death [8,29].

In our study, the risk of adverse hospital outcomes (ICU admissions, need of IMV and need of NIMV), increased with admission LUS scores between 8 to 24. The small number of patients presenting a score > 24 might explain the lack of correlation above this range. Also, the odds of adverse outcomes (in-hospital death, need for IMV, ICU admission) was almost four times higher in patients with LUS score ≥17 on admission than in those with a lower LUS score. A recent systematic review, including 11 studies, identified two 12-zone LUS score cut-offs, i.e., ≥15 [34] and ≥19.5 [20], as predictors of poor outcome (ventilation support, ICU admission, or 28-day mortality) in adults hospitalized with COVID-19 pneumonia [18]. Our data point in the same direction, towards a consensus for the LUS cut-off for prognosis and thus decision-making in COVID-19 pneumonia. For example, in patients with LUS scores of ≥17, the need for intensive care and access to NIMV and IMV should be anticipated.

A cut-off value of 15 for a 12-zone LUS score was an independent predictor of death among 402 patients admitted to three Chinese hospitals [23]. Similarly, a 12-zone LUS score cut-off ≥17 has been reported to be independently associated with in-hospital mortality among 37 hospitalized COVID-19 patients older than 65 years of age in Italy [35]. The systematic review previously mentioned [18] did not identify any LUS score cut-off being predictive of 28-day in-hospital mortality. However, this may be explained by the small sample sizes of the reported studies or absence of adjustment for comorbidities. In our study, the cut-off ≥17 was predictive of in-hospital mortality, irrespective of known comorbidities associated with an increased risk of death [32]. In a recent meta-analysis [29], the LUS score mean was 4.85 higher (95% CI 3.82–5.87) in non-survivors compared to survivors, ranging from 16.5 (SD5.8) [21] to 23 (SD 5) [36]. Lichter reported an optimal cut-off of >18 with a sensitivity of 62% and specificity of 74% in predicting 30 days mortality [22].

The difference between the scores of the first and second LUS examinations performed during hospitalization was not a good predictor of any of the adverse hospital outcomes, suggesting that repeating the LUS examination within 72 h of the first LUS does not provide any additional information for assessing patient prognosis. These results contrast with those of a study conducted among 30 patients in Germany, which found that changes in a LUS repeated three days after the initial LUS predicted a transfer to intensive care during hospitalization [37]. Compared to our study, their series of patients showed lower LUS scores at baseline (median 8; IQR 4–14) but higher proportions of ICU admission (27%) and in-hospital death (16%).

LUS is known to be operator-dependent [38]. In our study, the clinician LUS score was 2 points higher on average than the expert score. This difference might be partly due to an observer bias. Indeed, the clinicians who performed the bedside LUS were aware of the patient’s clinical condition, which could have influenced their LUS interpretation while the experts were blinded to the patient’s condition. A moderate inter-observer agreement for LUS has been found in other studies [17,39]. The rating also appears to vary between specialties, especially when grading B-line severity, with ER medical doctors scoring higher on average than radiologists or intensive care specialists [39]. We considered the inter-observer agreement between the trained clinicians and the off-site LUS experts as acceptable in our study. Moreover, this was further verified in most of the patients with a cut-off ≥17. This confirms that a short training period is sufficient to learn how to perform LUS correctly in patients hospitalized with COVID-19 pneumonia as long as a standardized LUS approach is used [38,40]. Furthermore, the operator variability could be addressed through the advancements in artificial intelligence (AI) applied to LUS. These developments have been demonstrated in assessing the extend of COVID-19 pneumonia [41] and diagnosing COVID-19 pneumonia compared to CT [42].

Our study has several strengths. All COVID-19 diagnoses were confirmed by RT-PCR, the diagnostic gold standard [43]. In addition, LUS examinations were systematically provided to all participants within the first 48 h after admission and repeated within the following 72 h, following a standardized protocol for image acquisition and scoring. Lastly, the LUS scores were rated by ultrasound experts, blinded to the patient’s clinical condition, thus excluding an observer bias.

Our study has also limitations. First, one limitation of our study arises from the use of the SpO_2_/FiO_2_ ratio, which serves as a proxy measure of the partial pressure of oxygen in arterial blood (PaO_2_)/FiO_2_ ratio. The performance of the LUS results might have been different if the PaO_2_/FiO_2_ ratio had been used as the reference to assess the severity of the patient’s respiratory condition. In addition, although we aimed to measure SpO_2_ and FiO_2_ at the time of the LUS examination, it was not always possible, and some measurements were taken within four hours. This may have contributed to the lack of correlation observed, though this time interval is short. Secondly, the study took place in the resource-constrained conditions of the pandemic. As such, the overwhelmed clinicians were unable to include all consecutive patients. Instead, we used random selection to include a feasible number of patients per day with the aim of minimizing selection bias. This procedure increased the duration of recruitment, encompassing the variation in the hospital and ICU admission criteria over time. Third, the results of this single-center study carried out in a university hospital in Spain, with a limited number of patients, may not be applicable to other centers and populations with different socioeconomic and demographic backgrounds, limiting the generalizability of the results. The 12-zone LUS score has been widely studied in assessing the severity of COVID-19 patients but it may still deserve further comparison to other scoring systems to be established as a reference standard [29]. Yet, the results of the study could have an added value in resource-limited settings where access to X-ray or CT scans is limited or non-existent.

High resolution chest computed tomography is considered the gold standard to evaluate the severity of patients with COVID-19 pneumonia [44] due to its high sensitivity, enabling the quantification of lung involvement [45]. However, the relative ease of use, ease of learning [17] and safety of LUS make this portable imaging technology particularly interesting for LMICs. Similar studies conducted in LMICs countries would provide evidence to support building the capacity of their healthcare professionals to use LUS in the management of COVID-19 [38]. Moreover, the advances in AI could be applied even in novice observers [42] and is a promising tool to estimate clinical severity [41]. Further studies are also needed to evaluate the use of LUS after the introduction of COVID-19 vaccines and the recent virus variants that have led to a reduced severity of COVID-19.

## 5. Conclusions

The 12-zone LUS score measured at the time of hospital admission has limited performance for identifying severe lung involvement in adult patients with COVID-19 pneumonia. However, a high admission LUS score was associated with a higher risk of adverse outcomes during hospitalization; and patients with a LUS over 17 had significantly higher mortality. Therefore, the admission LUS score could play a role in the clinical management of patients hospitalized with COVID-19 pneumonia. Repeating the LUS examination within 72 h after hospital admission did not introduce new information for predicting adverse outcomes in our patients.

## Figures and Tables

**Table 1:** Baseline characteristics of study participants, admission to intensive care unit, type of oxygenation, and hospital outcome.

Demographic Characteristics (*n* = 248)	*n*	%
Male	151	61.6
Age, median {IQR}	60	{51–73}
Comorbidities (*n* = 248)		
Hypertension	122	49.2
Dyslipidaemia	86	34.7
Diabetes	66	26.6
Obesity	47	19.0
Hypothyroidism	19	7.7
Chronic cardiopathy	15	6.0
Chronic renal disease (advanced FG < 30)	12	4.8
Chronic obstructive pulmonary disease *	11	4.4
Current smoker	11	4.4
Signs and symptoms at admission (*n* = 248)		
Cough	200	80.6
Fever	177	71.4
Shortness of breath	128	51.6
Fatigue	116	46.8
Diarrhea	80	32.3
Myalgia/arthralgia	69	27.8
Headache	64	25.8
Taste disorder	53	21.4
Sputum production	48	19.4
Smell disorder	43	17.3
Laboratory results (*n* = 177)	Median	{IQR}
Hemoglobin (g/dL)	13.6	{12.5–14.7}
White-cell count (10^3^/µL)	6.21	{4.8–8.3}
Absolute neutrophil count (10^3^/µL)	4.4	{12.5–14.7}
Absolute lymphocyte count (10^3^/µL)	1.0	{0.8–1.3}
Platelet count (10^3^/µL)	191	{151–236}
Creatinine (mg/dL)	0.9	{0.8–1.1}
C-reactive protein (mg/dL)	4.6	{2.8–9.4}
D-dimer (mcg/L)	570	{360–10,000}
B-type natriuretic peptide (pg/mL)	234	{88–870}
Interleukin 6 (pg/mL)	29.0	{12–79}
Chest X-ray at admission (*n* = 247)	*n*	%
Bilateral infiltrates	230	93.1
Interstitial pattern	122	49.4
Alveolar infiltrate	11	4.5
Opacities	12	4.9
Consolidations	6	2.4
Pleural effusion	1	0.4
Pneumothorax	1	0.4
Second chest X-ray carried out	179	72.5
Improvements compared to first	87	35.2
Chest computed tomography (*n* = 60)		
Ground-glass opacities	40	66.7
Focal changes	23	38.3
Thromboembolism	7	11.7
Reticular infiltrate pattern	5	8.3
Pleural effusion	3	5.0
Type of required oxygen therapy (*n* = 248)		
Nasal cannula (24–40%)	238	96.0
Venturi mask (24–80%)	126	50.8
Monaghan mask	77	31.0
Non-invasive mechanical ventilation	61	24.6
Invasive mechanical ventilation	23	9.3
Prone positioning	33	13.3
ICU admission and hospital outcome (*n* = 248)		
Not admitted to ICU	212	85.5
Number of deaths	5	2.4
Admitted to ICU	36	14.5
Number of deaths	8	22.2

IQR: Interquartile range; ICU: intensive care unit. * Not requiring home oxygen therapy.

**Table 2:** Expert lung ultrasound (LUS) scores upon admission and over the course of hospitalization, oxygen parameters, and vital signs at the time of LUS examination.

	All LUS (*n* = 657)		First LUS (*n* = 248)		Second LUS (*n* = 212)	
	*n* or Median	(%) or {IQR}	*n* or Median	(%) or {IQR}	*n* or Median	(%) or {IQR}
LUS score by expert	10	{7–15}	11	{7–15}	10	{7–14}
Oxygen parameters						
SPO_2_ (%)	97	{95–98}	97	{95–98}	97	{95–98}
FiO_2_ (%)	28	{24–40}	28	{26–36}	28	{24–50}
SpO_2_/FiO_2_	339	{235–400}	342	{270–380}	334	{195–398}
Patients with SpO_2_/FiO_2_ <315	282	(42.9)	96	(38.7)	99	(46.7)
SPO_2_ (%)	96	{95–98}	96	{95–98}	97	{96–98}
FiO_2_ (%)	50	{36–80}	40	{32–62}	50	{36–80}
SpO_2_/FiO_2_	196	{122–271}	242	{154–295}	192	{121–247}
Patients with SpO_2_/FiO_2_ <235	163	(24.8)	43	(17.3)	66	(31.1)
SPO_2_ (%)	97	{95–98}	96	{94–98}	97	{96–98}
FiO_2_ (%)	70	{50–85}	80	{50–81}	77	{50–90}
SpO_2_/FiO_2_	130	{112–192}	123	{116–190}	126	{105–192}
Vital signs						
Respiratory rate (breaths/min)	20	{18–24}	20	{18–24}	20	{18–24}
Temperature (°C)	36	{35–36}	36	{35–36}	36	{35–36}
Systolic blood pressure (mm Hg)	122	{110–135}	120	{109–132}	122	{112–138}
Diastolic blood pressure (mm Hg)	73	{66–81}	73	{66–80}	73	{65–81}

LUS: Lung ultrasound; IQR: Interquartile range; SpO_2_: Oxygen saturation: FiO_2_: Fraction of inspired oxygen.

**Table 3:** Logistic regression: prediction of adverse outcomes by lung ultrasound score ≥17.

LUS Score Cut-Off ≥17	Univariate Analysis				Multivariate Analysis ^1^			
	*n*	Odds Ratio	(95% CI)	*p*	*n*	Odds Ratio	(95% CI)	*p*
ICU admission	238	2.98	(1.22–7.26)	0.016	235	3.50	(1.37–8.94)	0.009
IMV	246	2.81	(1.05–7.46)	0.011	243	3.31	(1.19–9.13)	0.020
In-hospital death	248	3.45	(1.07–11.2)	0.038	234	5.31	(1.38–20.4)	0.015
NIMV	234	2.93	(1.42–6.09)	0.004	231	3.77	(1.69–8.40)	0.001
Combined adverse outcomes	238	3.07	(1.36–7.02)	0.008	235	3.88	(1.59–9.48)	0.003

LUS: lung ultrasound; ICU: intensive care unit (ICU); IMV: need for invasive mechanical ventilation; Combined adverse outcomes (in-hospital death or ICU admission or need for invasive mechanical ventilation); NIMV: need for non-invasive mechanical ventilation. ^1^ Adjusted for age, gender, hypertension, diabetes, and smoking status.

## Data Availability

Individual pseudo-anonymized participant data that underlie the results reported in this article (text, tables, figures, and appendices, with data dictionary) will be made available to others upon submission of a proposal. Requests will be reviewed and sharing of the data will follow the conditions required by all applicable laws and the possible prior signature of any necessary agreement, in accordance with the legal framework set forth by Médecins Sans Frontières (MSF) data sharing policy, which ensures that all security, legal, and ethical concerns are addressed. (https://www.msf.org/sites/msf.org/files/msf_data_sharing_policycontact_infoannexes_final.pdf, accessed on 1 March 2024). For data access and additional related documents such as the study protocol readers may contact Robert Nsaibirni, Data Protection and Compliance Officer (robert.nsaibirni@epicentre.msf.org).

## Supplementary Materials

The following supporting information can be downloaded at: https://www.mdpi.com/article/10.3390/jcm13113282/s1, Table S1: Sensitivity and Specificity of various cut-off values for the expert lung ultrasound scores at admission, using the SpO_2_/FiO_2_ ratio (<315 versus ≥315) as reference. Table S2. Univariate logistic regression models assessing different cut-off values of the lung ultrasound score to predict adverse outcomes; Table S3. Area under the ROC curve (AUC), sensitivity, specificity, positive and negative predictive values (with 95% CI) of a LUS score ≥ 17 to identify any of the adverse outcomes ((in-hospital death, ICU admission, need for IMV); Figure S1. Scatter plots of SpO_2_/FiO_2_ ratio or lung ultrasound (LUS) score upon admission versus adverse outcomes, with locally weighted scatter plot smoothing (LOWESS) curve. 1A: In-hospital death vs. SpO_2_/FiO_2_; 1B: In-hospital Death vs. LUS score; 2A: ICU admission vs. SpO_2_/FiO_2_; 2B: ICU admission vs. LUS score; 3A: Invasive mechanical ventilation vs. SpO_2_/FiO_2_; 3B: Invasive mechanical ventilation vs. LUS score; 4A: Combined adverse outcomes (in-hospital death or ICU admission or need for invasive mechanical ventilation) vs. SpO_2_/FiO_2_; 4B: Combined adverse outcomes vs. LUS score. Figure S2. Scatter plots of SpO_2_/FiO_2_ ratio or lung ultrasound (LUS) score upon admission versus the need for non-invasive mechanical ventilation (NIMV), with locally weighted scatter plot smoothing (LOWESS) curve. Left: NIMV vs. SpO_2_/FiO_2_; Right: NIMV vs. LUS score; Figure S3. Scatter plots of the difference between the scores of the first and the second lung ultrasound (LUS) examinations versus adverse outcomes, with locally weighted scatter plot smoothing (LOWESS) curve; Figure S4. Bland-Altman analysis between normalised inverted LUS score and normalised SpO2/FiO2 in the presence of any of the adverse outcomes (in-hospital death, ICU admission, need for IMV).
