# Supplementary material for: Evaluation of a Lung Ultrasound Score in Hospitalized Adult Patients with COVID-19 in Barcelona, Spain
Source: J Clin Med. 2024 Jun 2;13(11):3282. doi: 10.3390/jcm13113282 (PMC11172895; doi:10.3390/jcm13113282)
Supplement: Supplementary file 1 [file jcm-13-03282-s001.zip › jcm-2947838-supplementary.pdf]

SUPPLEMENTARY TABLES AND FIGURES

Table S1. Sensitivity and Specificity of various cut-off values for the expert lung ultrasound scores at admission, using the SpO2/FiO2 ratio (<315 versus ≥315) as gold standard.

LUS score	Patients (N=248)			LUS score cut-off	Sensitivity (%)	Specificity (%)	Correctly classified (%)
	n	(%)	Cumulative %				
**0**	1	(0.4)	0.4	≥0	100.0	0.0	38.7
**1**	3	(1.2)	1.6	≥1	100.0	0.7	39.1
**2**	4	(1.6)	3.2	≥2	100.0	2.6	40.3
**3**	4	(1.6)	4.8	≥3	100.0	5.3	41.9
**4**	10	(4.0)	8.8	≥4	100.0	7.9	43.6
**5**	14	(5.6)	14.5	≥5	99.0	13.8	46.8
**6**	16	(6.4)	20.9	≥6	94.8	20.4	49.2
**7**	13	(5.2)	26.2	≥7	93.8	30.3	54.8
**8**	16	(6.4)	32.6	≥8	89.6	36.2	56.9
**9**	20	(8.1)	40.7	≥9	84.4	43.4	59.3
**10**	23	(9.3)	50.0	≥10	77.1	52.0	61.7
**11**	22	(8.9)	58.8	≥11	69.8	62.5	65.3
**12**	9	(3.6)	62.5	≥12	59.4	70.4	66.1
**13**	15	(6.1)	68.5	≥13	56.3	74.3	67.3
**14**	14	(5.6)	74.1	≥14	46.9	78.3	66.1
**15**	13	(5.2)	79.4	≥15	38.5	82.2	65.3
**16**	10	(4.0)	83.4	≥16	28.1	84.2	62.5
**17**	4	(1.6)	85.0	≥17	24.0	88.2	63.3
**18**	6	(2.4)	87.5	≥18	22.9	90.1	64.1
**19**	7	(2.8)	90.3	≥19	19.8	92.1	64.1
**20**	8	(3.2)	93.5	≥20	17.7	95.4	65.3
**21**	1	(0.4)	93.9	≥21	11.5	96.7	63.7
**22**	5	(2.0)	95.9	≥22	11.5	97.4	64.1
**23**	1	(0.4)	96.3	≥23	8.3	98.7	63.7
**24**	3	(1.2)	97.5	≥24	7.3	98.7	63.3
**25**	2	(0.8)	98.3	≥25	4.2	98.7	62.1
**26**	1	(0.4)	98.7	≥26	3.1	99.3	62.1
**27**	2	(0.8)	99.6	≥27	3.1	100.0	62.5
**33**	1	(0.4)	100	≥33	1.0	100.0	61.7

LUS: lung ultrasound.

Table S2. Univariate logistic regression models assessing different cut-off values of the lung ultrasound score to predict adverse outcomes.

Adverse event	LUS score cut-off	Odds ratio	(95% CI)		P
**ICU admission (n=238)**	≥7	3.61	(0.82-	15.8)	0.089
	≥14	2.89	(1.26-	6.60)	**0.012**
	≥15	2.32	(1.01-	5.36)	0.050
	≥16	2.61	(1.10-	6.16)	0.029
	≥17	2.98	(1.22-	7.26)	**0.016**
	≥18	2.80	(1.12-	7.04)	**0.028**
	≥19	2.89	(1.10-	7.58)	**0.031**
**IMV (n=238)**	≥7	5.71	(0.75-	43.6)	0.093
	≥14	3.43	(1.25-	9.40)	**0.017**
	≥15	3.57	(1.31-	9.70)	**0.013**
	≥16	3.68	(1.34-	10.1)	**0.011**
	≥17	3.85	(1.37-	10.8)	**0.011**
	≥18	4.46	(1.58-	12.6)	**0.005**
	≥19	3.20	(1.14-	9.00)	**0.027**
**In-hospital death (n=248)**	≥7	0.88	(0.23-	3.31)	0.848
	≥14	2.69	(1.55-	8.30)	0.084
	≥15	2.62	(0.87-	8.10)	0.095
	≥16	2.57	(0.84-	8.22)	0.112
	≥17	3.45	(1.06-	11.2)	**0.038**
	≥18	3.96	(1.22-	12.9)	**0.022**
	≥19	3.42	(0.99-	11.9)	0.053
**Combined outcomes (n=238)**	≥7	2.07	(0.69-	6.20)	0.194
	≥14	2.89	(1.36-	6.17)	**0.006**
	≥15	2.56	(1.19-	5.53)	**0.017**
	≥16	2.57	(1.16-	5.70)	**0.020**
	≥17	3.07	(1.35-	7.02)	**0.008**
	≥18	3.01	(1.29-	7.05)	**0.011**
	≥19	3.27	(1.35-	7.96)	**0.009**
**NIMV (n=237)**	≥7	2.23	(0.89-	5.60)	0.086
	≥14	2.35	(1.24-	4.45)	**0.008**
	≥15	2.21	(1.14-	4.27)	**0.018**
	≥16	2.53	(1.27-	5.02)	**0.008**
	≥17	2.94	(1.42-	6.09)	**0.004**
	≥18	2.31	(1.08-	4.95)	**0.031**
	≥19	2.61	(1.15-	5.95)	**0.022**

LUS: lung ultrasound; ICU: intensive care unit; IMV: need for invasive mechanical ventilation; Combined outcomes (in-hospital death or ICU admission or need for invasive mechanical ventilation); NIMV: need for non-invasive mechanical ventilation.

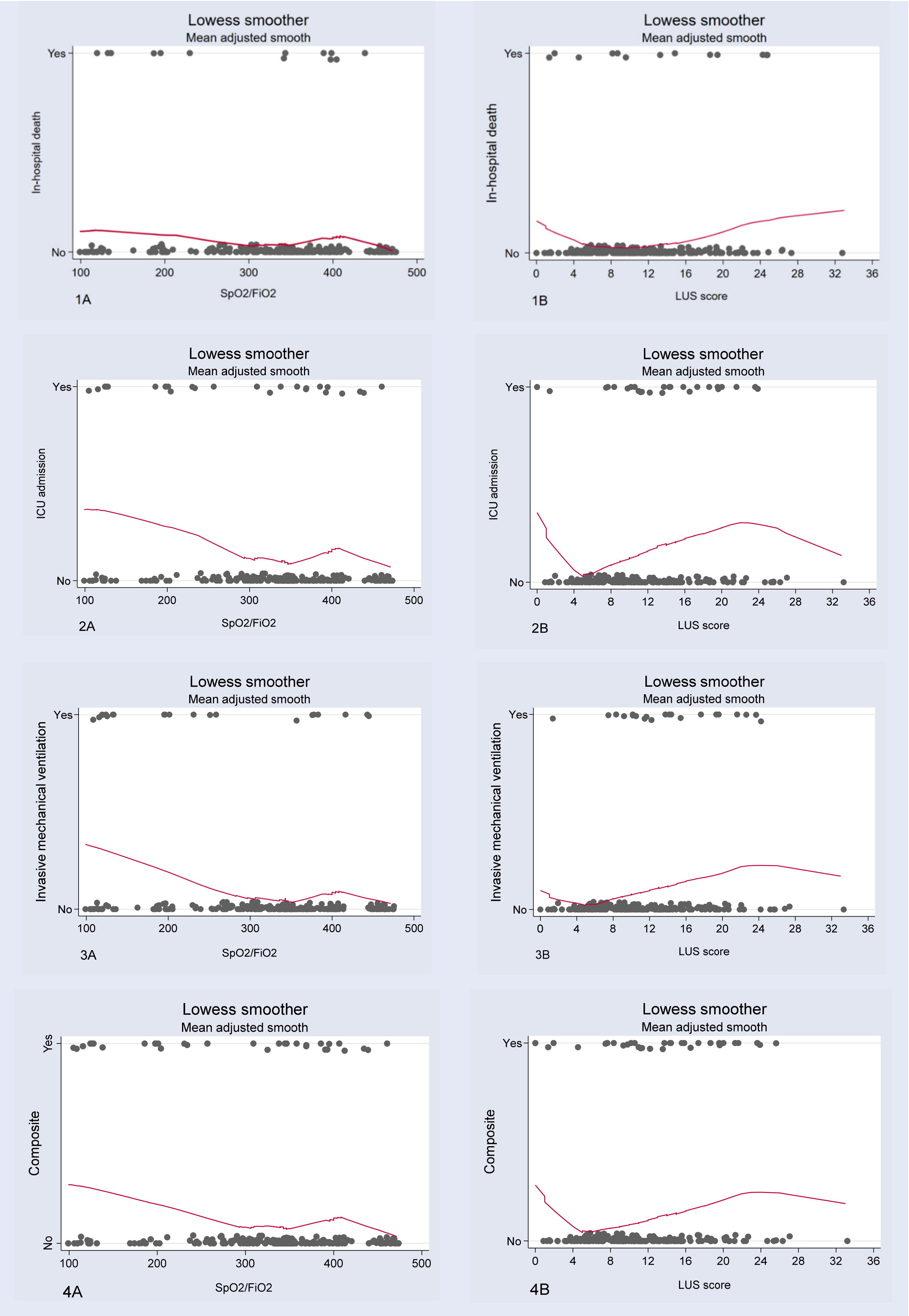

Figure S1. Scatter plots of SpO2/FiO2 ratio or lung ultrasound (LUS) score upon admission *versus* adverse outcomes, with locally weighted scatter plot smoothing (LOWESS) curve. ***1A****:* In-hospital death vs SpO2/FiO2; **1B**: In-hospital Death vs LUS score; **2A:** ICU admission vs SpO2/FiO2; **2B:** ICU admission vs LUS score; **3A:** Invasive mechanical ventilation vs

SpO2/FiO2; **3B:** Invasive mechanical ventilation vs LUS score; **4A:** Combined outcomes (in-hospital death or ICU admission or need for invasive mechanical ventilation) vs SpO2/FiO2; **4B:** Combines outcomes vs LUS score.

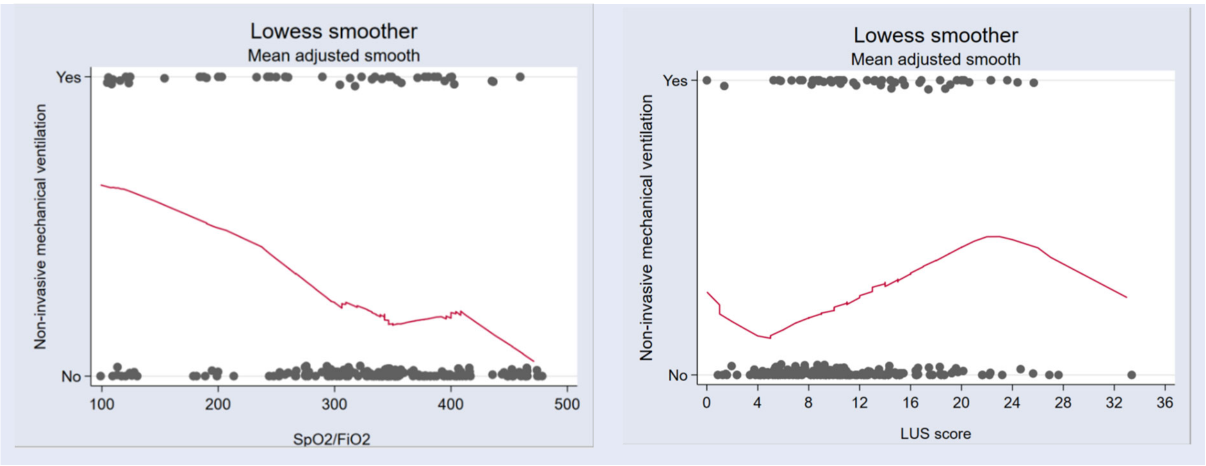

Figure S2. Scatter plots of SpO2/FiO2 ratio or lung ultrasound (LUS) score upon admission versus the need for non-invasive mechanical ventilation (NIMV), with locally weighted scatter plot smoothing (LOWESS) curve. **Left**: NIMV vs SpO2/FiO2; **Right**: NIMV vs LUS score.

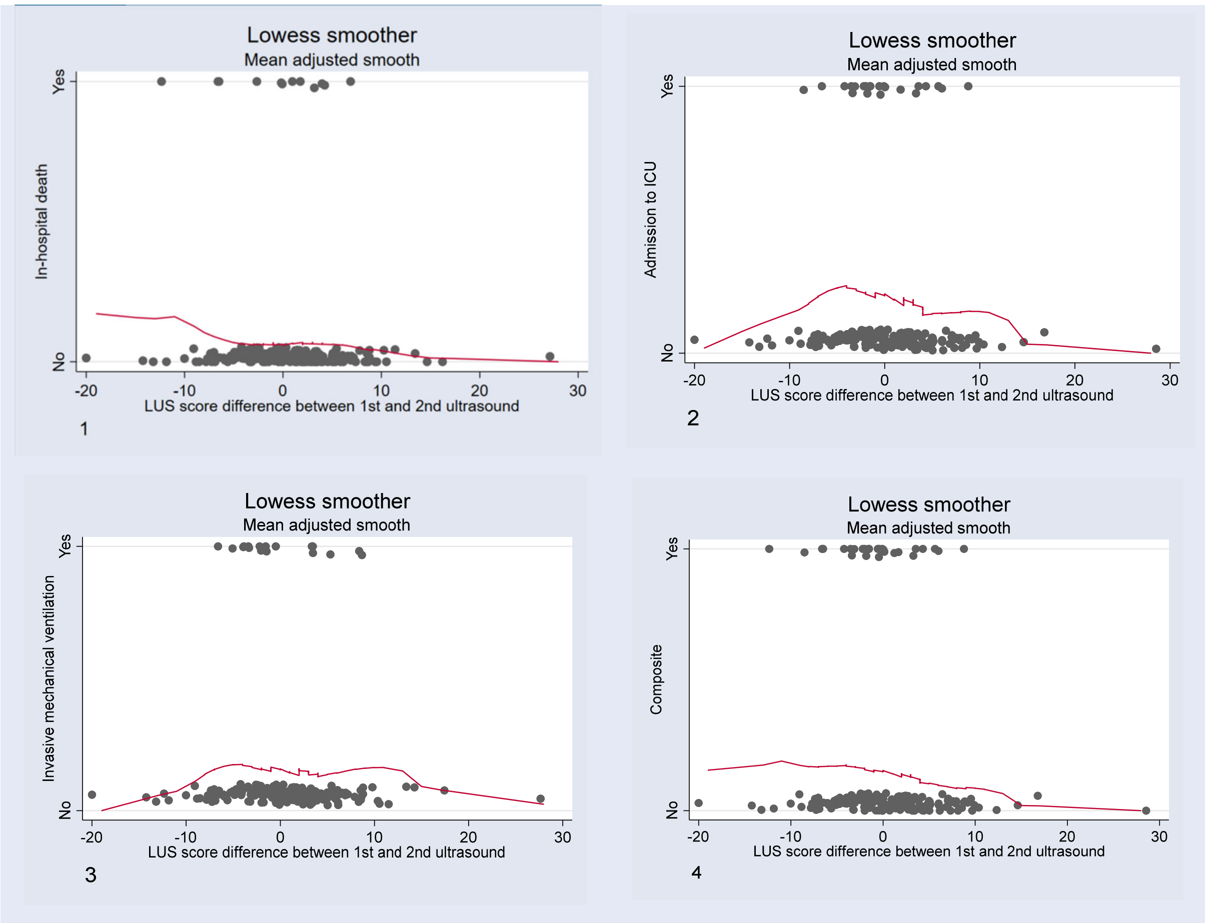

Figure S3. Scatter plots of the difference between the scores of the first and the second lung ultrasound (LUS) examinations versus adverse outcomes, with locally weighted scatter plot smoothing (LOWESS) curve. **1**: In-hospital death; **2:** ICU admission; **3:** Need for invasive mechanical ventilation; **4:** Combined (in-hospital death or ICU admission or need for invasive mechanical ventilation).

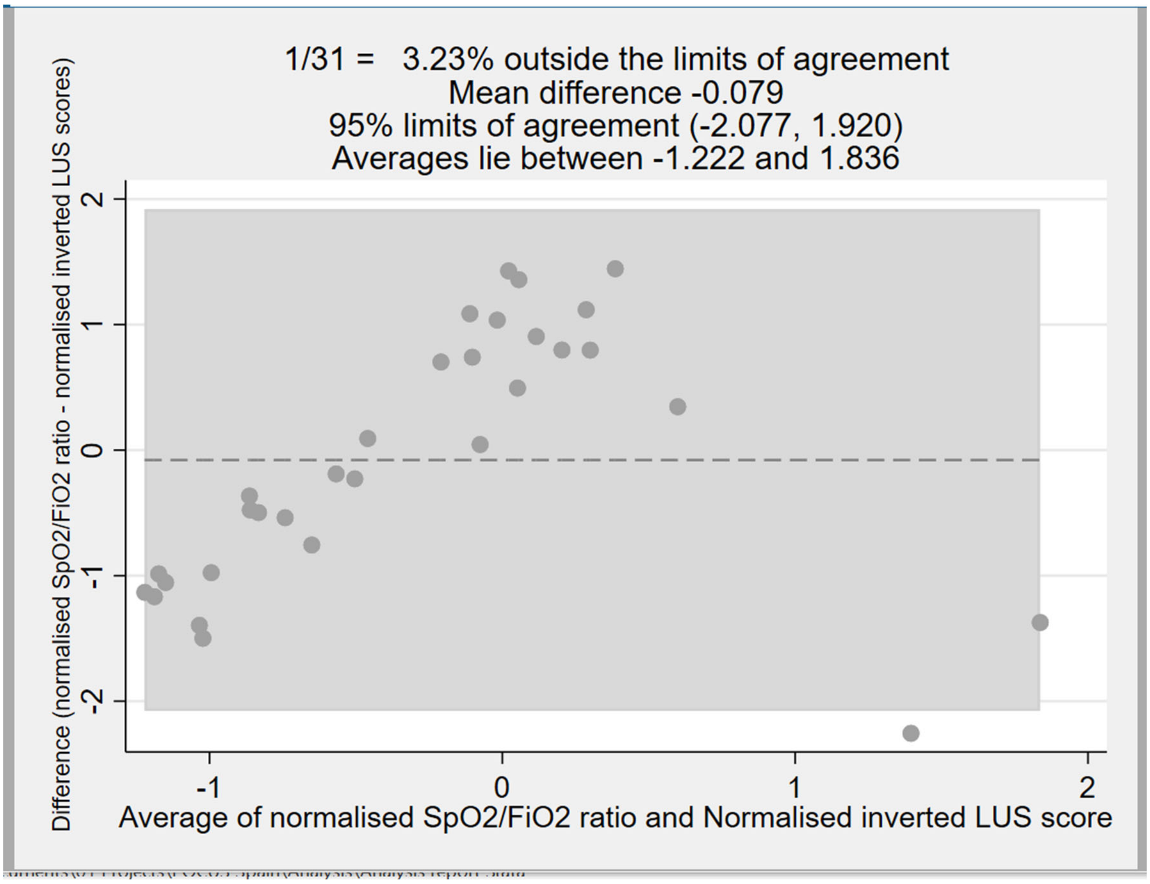

Figure S4. Bland-Altman analysis between normalised inverted LUS score and normalised SpO2/FiO2 in the presence of any of the adverse outcomes (in-hospital death, ICU admission, need for IMV).

We observed that as the severity of the clinical situation increases (and the average of normalised SpO2/FiO2 and inverted LUS score decreases), the normalised inverted LUS scores tend to be higher than the normalised SpO2/FiO2 ratio in the presence of any of the adverse outcomes. Conversely, in milder clinical situations, the normalised SpO2/FiO2 ratio tends to exceed the LUS scores. The mean difference between these measures is -0.08, with only one data point (3.23%) falling outside the limits of agreement.

The Spearman's rank correlation coefficient between the normalized SpO2/FiO2 ratio and the normalised inverted LUS score was 0.36 ($p<0.001$). When restricting to patients who presented at least one adverse outcome (n=31), this coefficient strengthens to 0.41 (p=0,02).

Table S3. Area under the ROC curve (AUC), sensitivity, specificity, positive and negative predictive values (with 95% CI) of a LUS score ≥ 17 to identify any of the adverse outcomes ((in-hospital death, ICU admission, need for IMV).

**LUS score cut-off ≥17**		**AUC**	**Sensitivity (%)**	**Specificity (%)**	**Positive Predictive Value (%)**	**Negative Predictive Value (%)**
	**N**	**95% CI**	**95% CI**	**95% CI**	**95% CI**	**95% CI**

ICU admission	238	0.62	22	86	25	85
		(0.53-0.72)	(17-27)	(83-91)	(20-30)	(80-89)
IMV	246	0.66	17	92	30	85
		(0.55-0.77)	(12-22)	(89-96)	(25-36)	(80-89)
In-hospital death	248	0.58	12	96	38	85
		(0.37-0.79)	(8-16)	(94-99)	(32-45)	(80-89)
NIMV	234	0.61	39	78	26	87
		(0.53-0.69)	(33-45)	(73-83)	(21-32)	(82-91)
Combined adverse outcomes	238	0.63	27	89	34	85
		(0.52-0.74)	(21-32)	(85-93)	(28-40)	(81-90)
